# The application of virtual reality meditation and mind–body exercises among older adults

**DOI:** 10.3389/fpsyg.2024.1303880

**Published:** 2024-03-19

**Authors:** Dong Gao, Yuqin Su, Xing Zhang, Hansen Li, Hongcheng Luo

**Affiliations:** ^1^College of Physical Education, Chongqing University of Posts and Telecommunications, Chongqing, China; ^2^Institute of Sports Science, College of Physical Education, Southwest University, Chongqing, China; ^3^Department of Physical Education and Sport, Faculty of Sport Sciences, University of Granada, Granada, Spain; ^4^School of Physical Education, Xichang University, Xichang, China

**Keywords:** virtual reality, public health, mindfulness, meditation, elderly, mind–body exercises

## Abstract

Virtual reality (VR)-based mindfulness is a promising method to improve the health of older adults. Therefore, many attempts have been made to explore the application of VR-based mindfulness, such as VR meditation and mind–body exercises, in older adults. Generally, current studies indicate the heavy reliance on apparatus for implementing VR-based mindfulness interventions. In VR meditation, the crucial apparatus is VR headsets. In VR mind–body exercises, three essential components are required: motion capture sensors, main consoles, and display screens. In the aspect of health promotion, VR meditation is an effective method for improving mental health, pain, and quality of life in older adults. VR mind–body exercises contribute to increasing the mental health and physical function of older adults. Furthermore, VR mind–body exercises may be combined with other forms of exercise as a mixed method to promote the health of older adults. VR-based mindfulness interventions enhance the meditation and mind–body exercises experience for older adults while improving accessibility. However, their implementation still encounters a series of challenges, such as cost, technical anxiety, and apparatus-related issues. Additionally, we recommend future research to examine the optimal exercise dose for VR mind–body exercises to maximize their health benefits.

## 1 Introduction

In recent decades, the global population has experienced unprecedented demographic shifts due to the aging population (Zhao et al., 2022). As this trend continues, it is projected that by 2050, approximately 22% of the world’s population will consist of individuals aged 65 and older (Zenebe et al., 2021; Zhao et al., 2022). The aging process involves a natural decline in physiological functions, including reduced performance in various organ systems, and a decreased ability to withstand physical, cognitive, and psychological stressors (Norman et al., 2021). These age-related changes increase the risk of health problems among older adults. In the context of an aging population, this leads to substantial economic burdens and an increased demand for healthcare services on a global scale (Collado-Mateo et al., 2021; Norman et al., 2021). Therefore, there is an urgent need to develop various health promotion strategies to enhance the health of older adults.

Mindfulness practice is the intentional cultivation of non-judgmental awareness and presence in the present moment, often achieved through meditation and mind–body exercises (Gupta, 2021). While rooted in Buddhist traditions, mindfulness practice has gained increasing attention from researchers in medicine and psychology (Scheepers et al., 2020; Fendel et al., 2021). In the past decades, researchers have identified various health benefits associated with mindfulness practice, such as improving mental health problems (Enkema et al., 2020), life quality (Valikhani et al., 2020), pain (Ngamkham et al., 2019), cardiovascular disease (Conversano et al., 2021), and other diseases (Giulietti et al., 2023; Naude et al., 2023). At present, mindfulness-based intervention has been widely implemented in various secular populations: spanning from pre-school children to older adults (Jamieson and Tuckey, 2017; Carsley et al., 2018; Odgers et al., 2020; Kriakous et al., 2021; Zhang et al., 2021). However, the implication of mindfulness practice still faces a range of challenges. On one hand, mindfulness practice requires conscious effort and can be challenging to maintain, particularly for novice meditators who may already be using substantial cognitive capacity to enhance their self-regulatory skills (Seabrook et al., 2020). On the other hand, many factors can impact the implementation of mindfulness practice, such as environment, attention, cognition, physiology, emotional well-being, and social relationships (Seabrook et al., 2020; Li, 2022; Ma et al., 2023). Therefore, traditional mindfulness practice requires under the guidance of professional trainers in specific locations (e.g., meditation center and hospital) (Ma et al., 2023). However, this method limits the accessibility of mindfulness practice, particularly for older adults.

**Figure 1:**
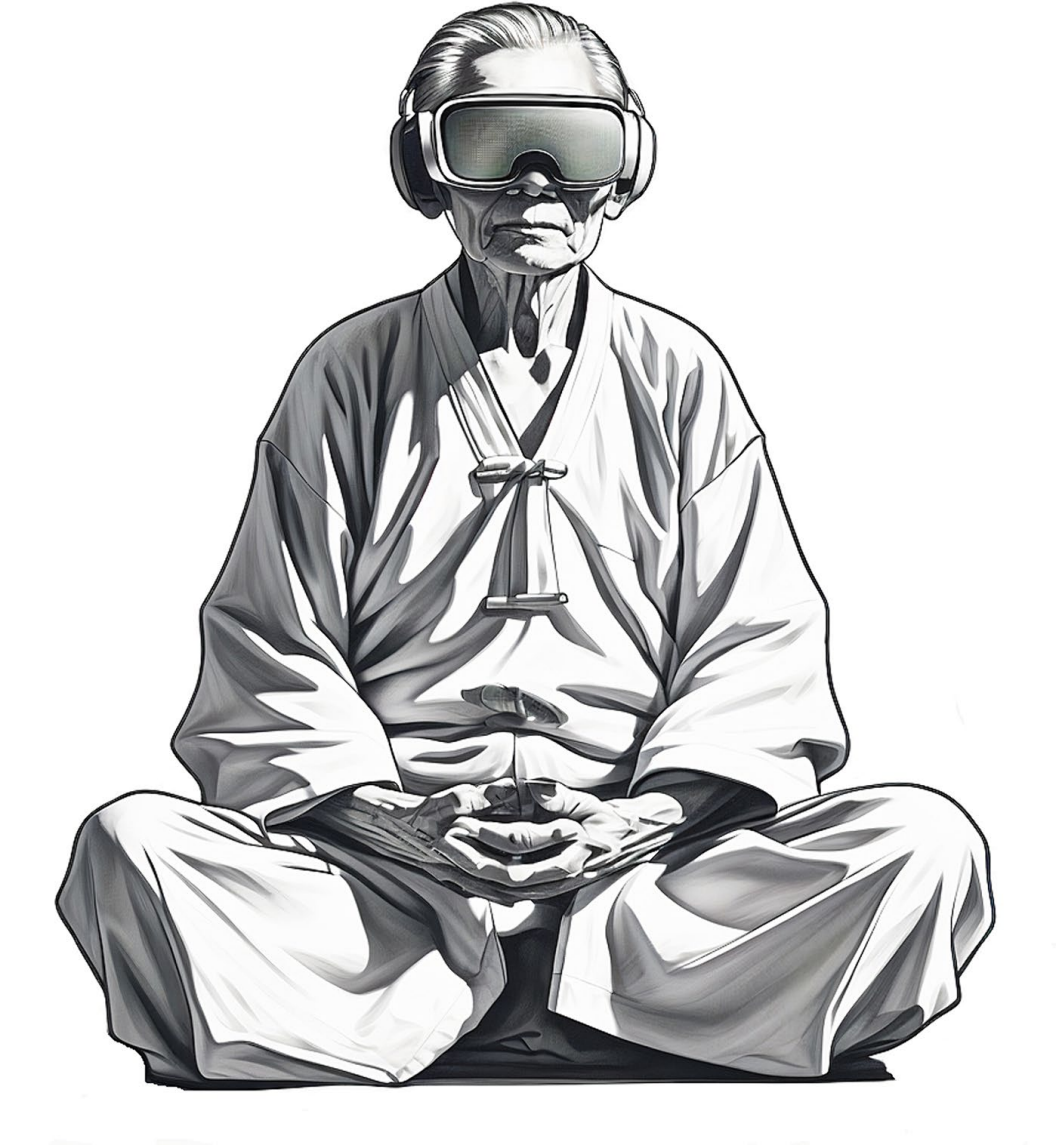
VR-based mindfulness.

In order to improve the accessibility of mindfulness practice, extensive exploration has been undertaken (Hendrixson, 2020; May and Maurin, 2021; Wu et al., 2022). One promising solution is the application of virtual reality (VR) technology in mindfulness practice. VR technology allows individuals to engage with multisensory experiences in immersive 3D environments, potentially enhancing their mindfulness practice (Lee et al., 2023). To date, some studies have examined the application of VR technology to meditation and mind–body exercises for older adults (Chen et al., 2020; Hendrixson, 2020). However, comprehensive information on the implementation of VR meditation and mind–body exercises in older adults is still lacking (Figure 1). Therefore, we conducted this review based on published literature to address the following questions:

- How are VR meditation and mind–body exercises implemented in older adults?
- What is the impact of VR meditation and mind–body exercises on the health of older adults?
- What are the strengths and challenges of implementing VR meditation and mind–body exercises in older adults?

## 2 VR meditation

### 2.1 Implementation

#### 2.1.1 Apparatus

VR headsets are a crucial component of VR meditation. Typically, a VR headset comprises a screen and lenses projecting the virtual environment directly in front of the user’s eyes, elevating the meditation experience (Li et al., 2021). Additionally, VR headsets often feature built-in headphones or speakers to further enrich the user’s multisensory immersive experience. Traditionally, VR headsets operate as output devices, necessitating a connection to main consoles (e.g., Xbox, PS, PC, and smartphone) for generating the intervention environment (Dzardanova and Kasapakis, 2022). However, it’s essential to note that main consoles are not always obligatory components. With the advancement of VR technology, all-in-one VR headsets (e.g., PICO 4 VR Headset, HTC Vive Focus 3 Headset, Meta Quest Pro VR Headset) have been developed (Wang et al., 2023). This type of device is equipped with a built-in processor, offering independent processing, input, and output functions (Al Arafat et al., 2021). Hence, there is no need for connections to a main console, eliminating wired constraints. Additionally, in order to reduce the cost of VR interventions, some researchers have tried to use mobile phone VR headsets (e.g., the Destek V5 headset) (Trueba et al., 2023a,b). These devices are relatively inexpensive and require users to insert their smartphones into the headset to experience virtual reality content (Huang and Wang, 2021).

#### 2.1.2 Implementation process

The implementation process of VR meditation is as follows:

- Preparation phase: Firstly, researchers formulate an intervention plan. Subsequently, researchers prepare VR apparatuses and select suitable videos based their design. Finally, researchers provide participants with guidance and instructions related to VR meditation.
- Implementation phase: First, participants wear VR headsets and personalize settings to enhance their meditation experience. Subsequently, participants follow the instructions for meditation practice. Finally, after completing meditation practice, participants remove VR headsets. Throughout the VR meditation process, supervisors in the real world provide support and guidance to ensure participant safety.
- Testing and evaluation phase: Researchers will request participants to complete a series of questionnaires and tests at specific stages of a study, such as before and after meditation intervention, depending on their study design.

To minimize the risk of potential adverse events such as motion sickness and eye strain among older adults, VR meditation interventions are typically set for 5–15 min (Sarkar et al., 2022; Cinalioglu et al., 2023a,b). Furthermore, the implementation of VR meditation in older adults generally follows two methods: remote and in-person. In the in-person method, participants engage in VR meditation at a location prepared by the researchers, such as a hospital (Cinalioglu et al., 2023a). In the remote method, participants receive the necessary apparatus and online guidance to practice VR meditation in their own homes. Some studies also conducted post-intervention interviews with participants to gather insights into their experiences, emotions, and feedback regarding VR meditation (Hendrixson, 2020).

### 2.2 Benefits

In the past decades, meditation has been considered an effective method for improving the physical and mental health of older adults (Weber et al., 2020; Reangsing et al., 2021). In VR meditation, similar findings were also observed. In terms of mental health, studies by Cinalioglu et al. (2023b) and Trueba et al. (2023a,b) claimed that VR meditation can reduce stress and alleviate negative mood symptoms (e.g., anxiety and depression) in older adults. However, these three studies only published abstracts in The American Journal of Geriatric Psychiatry, and detailed information is unavailable. Additionally, Sarkar et al. (2022) explored the impact of VR meditation on older adults with knee osteoarthritis and found a significant reduction in negative emotions following the intervention, with this effect still noticeable after 24–48 h. Although not statistically significant, Sarkar et al. (2022) also observed an improvement in positive emotions among older adults after VR meditation. In terms of physical health, Sarkar et al. (2022) found that a 10-min VR meditation is a safe and feasible intervention for improving chronic pain, both overall and knee-specific, in older adults with osteoarthritis. Another study examining the impact of VR meditation on older adults’ quality of life found that a 3-week VR meditation program effectively enhanced overall quality of life (Hendrixson, 2020). This finding may imply an improvement in overall physical and mental health conditions.

One perspective suggests that VR meditation may be more effective than traditional meditation because it enhances the meditation experience (Ma et al., 2023). However, there is limited research comparing the health benefits of VR meditation to traditional meditation. One reason for the limited research might be that VR meditation is a new and emerging research topic, and many related studies may still be in progress. Additionally, researchers within the field of VR meditation may have diverse research priorities, and their focus not be solely concentrated on one theme. Therefore, it remains unclear whether the enhanced meditation experience translates into greater health benefits for older adults. Given this, we recommend future research to explore the most effective meditation methods. Furthermore, future studies should identify potential variations in the health benefits of VR meditation among different populations, such as older adults vs. younger individuals, males vs. females, and the general public vs. specific groups.

### 2.3 Strength

The strengths of VR meditation are as follows:

- Meditation experience: VR meditation offers a more immersive and engaging experience compared to traditional meditation. The use of virtual environments and sensory stimuli can enhance the sense of relaxation and concentration during meditation. In the study conducted by Sarkar et al. (2022), participants expressed that VR meditation enhanced their meditation experience, with 94.7% of participants expressing their willingness to engage in VR meditation again.
- Accessibility: VR meditation can be easily accessed and practiced from the comfort of one’s own home. For instance, in the research by Cinalioglu et al. (2023a), participants were allowed to receive remote guidance and engage in VR meditation interventions within their homes. This accessibility is advantageous for older adults with limited mobility.
- Personalization: VR meditation programs can be customized to cater to individual needs and intervention objectives. This customization is advantageous for enhancing the personalization and specificity of meditation.

### 2.4 Implementation challenge

The implementation challenges of VR meditation are as follows:

- Cost: VR meditation requires specialized apparatus, such as a main console + VR headset or an all-in-one VR headset. The prices for all-in-one VR headsets typically range between $200 and $500, with high-end devices featuring advanced display technology, tracking systems, and processors costing more, such as the Pimax Crystal VR headsets. Traditional VR headsets are generally more affordable, ranging in price from $100 to $300, but they require connection to a main console. The cost of these devices can be a barrier to widespread adoption, particularly among older adults in developing countries. Fortunately, some researchers are exploring low-cost VR meditation intervention solutions (Trueba et al., 2023a,b). They utilized free VR mobile apps and the Destek V5 headset (a mobile phone VR headset priced at a few tens of dollars) to investigate the effectiveness of VR meditation in improving the mental health of older adults. Their future findings could potentially benefit economically disadvantaged populations.
- Technical Anxiety: Some older adults may express concerns about using VR devices due to a lack of familiarity (Trueba et al., 2023a,b). Training and technical support may be necessary to alleviate their technological anxiety.
- Simulator sickness: Prolonged use of VR headsets can lead to discomfort or sickness. Current studies typically control the duration of VR meditation. Meanwhile, Sarkar et al. (2022) indicated that older adults generally reported scores of 0 (none) or 1 (slight) for each symptoms on the simulator sickness questionnaire, which measures the severity of 16 symptoms (such as headache, eye strain, and blurred vision) experienced during VR interventions (Sevinc and Berkman, 2020). Nevertheless, some older adults still reported experiencing severe migraines the day after the intervention (Sarkar et al., 2022).
- Apparatus: Certain apparatuses may lack stability and could malfunction, leading to interruptions during interventions (Trueba et al., 2023a,b). Additionally, compatibility issues between VR videos and devices may affect intervention implementation (Trueba et al., 2023a,b).
- Video Content: Most VR videos are primarily designed for entertainment purposes and may not be suitable for VR meditation (Trueba et al., 2023a,b). Furthermore, language support for the videos may also pose a potential issue (some languages may not be supported) (Trueba et al., 2023a,b).

## 3 VR mind–body exercises

### 3.1 Implementation

#### 3.1.1 Apparatus

Traditionally, implementing VR mind–body exercises requires hardware support such as motion capture sensors, main consoles, and display screens (Kim et al., 2013; Rahman, 2023). In VR mind–body exercises, motion capture sensors are utilized to capture and monitor participants’ physical movements through cameras, infrared depth sensors, and various other sensors (Kim et al., 2013). Main consoles (e.g., Xbox, PS, or PC) are then used to link motion capture sensors, acquire motion capture-related data, and establish the VR environment (Kim et al., 2013). Display screens play a crucial role in presenting the virtual environment, showcasing visual elements related to movements and activities like exercise instructions, environmental backgrounds, and virtual participants (Rahman, 2023). Simultaneously, display screens serve as tools for providing feedback, enabling participants to observe their movements and actions, thereby facilitating self-monitoring and improvement (Rahman, 2023).

One thing to note is that current studies typically utilize display screens to present the VR environment when investigating the impacts of VR mind–body exercises on older people (Hsieh et al., 2019; Rahman, 2023). Wearable devices, such as standalone VR headsets and all-in-one VR headsets, are currently seldom employed. This may be associated with controlling research risks. In VR mind–body exercise interventions, older adults participate in a series of physical activities, and the potential motion sickness induced by using VR headsets may elevate the risk of exercise-related injuries (Cucher et al., 2023).

#### 3.1.2 Implementation process

The implementation process of VR meditation is as follows:

- Preparation phase: Firstly, researchers prepare VR apparatuses and set them up in a suitable location. Subsequently, researchers prepare the necessary software for VR mind–body exercises and formulate the intervention plan. Finally, researchers provide participants with guidance and instructions related to VR mind–body exercises.
- Implementation phase: Firstly, participants follow researchers’ instructions for warm-up. Subsequently, they engage in mind–body exercises guided by a VR system. Finally, under the guidance of the researchers, participants completed relaxation activities after the intervention. Throughout the VR meditation process, in addition to the virtual coach, medical personnel/researchers/coaches are typically present to monitor and support participants as needed.
- Testing and evaluation phase: Researchers will request participants to complete a series of questionnaires and tests at specific stages of a study, such as before and after meditation intervention, depending on their study design.

In VR mind–body exercises, to enhance the quality of motion capture and ensure participant comfort during viewing, motion capture sensors and display screens are positioned at appropriate heights (approximately 1 m above the ground) and distances (approximately 2–2.5 meters from the participants) (Hsieh et al., 2019; Rahman, 2023). VR mind–body exercises typically select slow, continuous movements such as Tai Chi and yoga (Kim et al., 2013; Rahman, 2023). A session typically lasts for 30–45 min, during which participants can view their own movements and receive motion scores (Kim et al., 2013; Hsieh et al., 2019; Rahman, 2023). Additionally, a virtual coach provides visual and verbal feedback on participants’ movements to ensure the correct execution of each motion (Hsieh et al., 2019; Chen et al., 2020; Rahman, 2023).

### 3.2 Benefits

Tai Chi and yoga are the main events in VR mind–body exercises. In terms of mental health, a study conducted by Hsieh et al. (2019) examined the impact of 6 months of VR Tai Chi on older adults with cognitive issues. Their research found that VR Tai Chi intervention significantly improved abstract thinking and judgment abilities in older adults. However, no significant outcomes were observed in long-term memory, short-term memory, attention, mental manipulation, orientation, language, drawing, and animal name fluency. Another study investigated the effects of VR Tai Chi on the mental health of older adults during the COVID-19 pandemic (Kim et al., 2022). Their research revealed that older adults experienced improvements in mindfulness and enjoyment after the VR intervention. In terms of physical health, Hsieh et al. (2019) found that VR Tai Chi improves the aerobic endurance, lower extremity endurance, balance, and gait speed of older adults. Similar findings were also observed in mixed VR mind–body exercises. The study by Kim et al. (2013) indicates that a combined intervention of VR Tai Chi and yoga improved hip muscle strength and balance in older adults. Furthermore, one study compared the health benefits of older adults participating in VR Tai Chi versus traditional Tai Chi (Chen et al., 2020). The results indicated that VR Tai Chi may have greater potential in improving balance function and strength.

In addition, some studies indicate that combining VR mind–body exercises with VR non-mind–body exercises can improve the health of older adults. For example, Padala et al. (2017) found that a combined intervention of VR yoga, strength training, and aerobic training improved the balance of older adults. Chao et al. (2015) used a similar mixed intervention and observed improvements in balance, mobility, and depression among older adults. These findings suggest that VR mind–body exercises may have the potential to combine with other forms of exercise.

In summary, current findings indicate that VR mind–body exercises and mixed exercises, including VR mind–body exercises, may positively impact the physical and mental health of older adults. This conclusion aligns with previous research based on traditional mind–body exercises (Zou et al., 2018; Wu et al., 2019). However, which intervention method is the most effective remains unclear, and further research is needed to explore this.

### 3.3 Strength

The strengths of VR meditation are as follows:

- Exercise quality: Some mind–body exercise events, such as the full Tai Chi sequence, can be challenging for older adults (Chen et al., 2020). VR interventions provide real-time visual and auditory feedback. Specifically, participants can see their own movements and postures during the intervention, receiving guidance from a virtual coach. This can help them improve the quality of their exercise.
- Accessibility: Learning certain mind–body exercises can be challenging, as mentioned above. In traditional mind–body exercises, older adults may need to visit specific locations for instruction from a coach. In VR interventions, participants can receive guidance from a virtual coach, which opens up the possibility of home-based exercise. Research by Kim et al. (2013) suggests that VR mind–body exercises can improve physical function among older adults in an unsupervised environment, where VR feedback can replace the role of a coach.
- Immersion: VR technology offers a highly immersive experience, making it easier for participants to engage and enjoy the exercise. According to Rahman (2023), most participants reported that VR mind–body exercises were enjoyable.

### 3.4 Implementation challenges

VR mind–body exercises implementation not only encounters the challenges mentioned in the previous section, such as the cost, technical anxiety, and apparatus, but also confronts the following issues:

- Exercise dose: VR mind–body exercises, such as Tai Chi and yoga, primarily consist of physical activities. Therefore, different exercise settings (e.g., intensity, frequency, and duration) may result in varying physical and mental health gains (Zhang et al., 2023a,b). However, there is limited evidence regarding the optimal exercise dose for VR mind–body exercises. Additionally, different older individuals may have different exercise dose requirements (Hsieh et al., 2019), and providing inappropriate exercise loads may lead to adverse effects. Thus, future research may need to further explore the optimal VR mind–body exercises dose under different conditions.
- Generalizability: Although research has identified the feasibility of seated Tai Chi exercise among frail older adults (Rahman, 2023), implementing VR mind–body exercise interventions in older individuals with functional impairments (e.g., mobility issues or memory problems) remains challenging due to the extensive amount of physical activity involved.

## 4 Limitation

When interpreting the findings of this study, it’s important to consider some limitations. Firstly, our research suggests the positive impact of VR meditation and mind–body exercises on the physical and mental health of older adults. However, due to limitations inherent in our study design and the limited number of existing studies, our findings were not supported by quantitative analyses. Therefore, we recommend that future research conducts relevant meta-analyses as more related studies emerge. Secondly, our study focused on the implementation and effects of VR meditation and mind–body exercises in older adults. Therefore, the findings of this study cannot be generalized to other populations. Finally, the current research only included the application of VR Tai Chi and yoga in older adults. Consequently, our findings cannot be extrapolated to other types of VR mind–body exercises, such as VR pilates and Qigong. We encourage future experimental studies to explore various types of VR mind–body exercises.

## 5 Conclusion

The purpose of the current research is to summarize the implementation and impact of VR meditation and mind–body exercises in older adults. Our study indicates that VR headsets are critical devices for implementing VR meditation (Trueba et al., 2023a,b). However, in VR mind–body exercises, such devices are rarely used, and researchers typically employ a combination of motion capture sensors, main consoles, and display screens (Kim et al., 2013; Rahman, 2023). This could be to mitigate the risk of motion sickness (Cucher et al., 2023). In terms of health promotion, VR meditation has been shown to improve mental health (Trueba et al., 2023a,b; Cinalioglu et al., 2023b), pain (Sarkar et al., 2022), and quality of life (Hendrixson, 2020) in older adults. VR mind–body exercises contribute to enhancing the mental health (Hsieh et al., 2019; Kim et al., 2022) and physical function (Chen et al., 2020) of older adults. Furthermore, VR-based mindfulness interventions not only improved the quality of meditation and mind–body exercises but also enhanced their accessibility (Kim et al., 2013; Cinalioglu et al., 2023a). However, the implementation of these interventions still faces challenges such as cost (Trueba et al., 2023a,b), simulator sickness (Sarkar et al., 2022), technical anxiety (Trueba et al., 2023a,b), and apparatus-related issues (Trueba et al., 2023a,b). Moreover, despite the positive impacts of VR mind–body exercises on the health of older adults observed in current research, the optimal exercise dose remains unclear. Therefore, future research should focus on addressing the issue of the optimal dosage for VR interventions.

## Author contributions

DG: Writing – review & editing, Funding acquisition, Visualization. YS: Writing – review & editing, Visualization. XZ: Visualization, Writing – original draft, Writing – review & editing. HLi: Writing – original draft, Writing – review & editing. HLu: Supervision, Writing – original draft, Writing – review & editing.

## References

[ref1] Al Arafat A. Guo Z. Awad A. (2021). “VR-Spy: a side-channel attack on virtual key-logging in VR headsets” in 2021 IEEE Virtual Reality and 3D User Interfaces (VR) (New York: IEEE), 564–572. doi: 10.1109/VR50410.2021.00081

[ref2] Carsley D. Khoury B. Heath N. L. (2018). Effectiveness of mindfulness interventions for mental health in schools: a comprehensive meta-analysis. Mindfulness 9, 693–707. doi: 10.1007/s12671-017-0839-2

[ref3] Chao Y.-Y. Scherer Y. K. Montgomery C. A. Wu Y.-W. Lucke K. T. (2015). Physical and psychosocial effects of Wii fit exergames use in assisted living residents: a pilot study. Clin. Nurs. Res. 24, 589–603. doi: 10.1177/1054773814562880, PMID: 25488422

[ref4] Chen P.-J. Penn I.-W. Wei S.-H. Chuang L.-R. Sung W.-H. (2020). Augmented reality-assisted training with selected tai-chi movements improves balance control and increases lower limb muscle strength in older adults: a prospective randomized trial. J. Exerc. Sci. Fit. 18, 142–147. doi: 10.1016/j.jesf.2020.05.003, PMID: 32514277 PMC7265060

[ref5] Cinalioglu K. Lavín P. Bein M. Lesage M. Gruber J. Se J.. (2023a). Effects of virtual reality guided meditation in older adults: the protocol of a pilot randomized controlled trial. Front. Psychol. 14:1083219. doi: 10.3389/fpsyg.2023.1083219, PMID: 37575420 PMC10421698

[ref6] Cinalioglu K. Sekhon H. Rej S. (2023b). Effects of a virtual reality assisted mindfulness intervention in older adults. Am. J. Geriatr. Psychiatry 31, S133–S134. doi: 10.1016/j.jagp.2022.12.184

[ref7] Collado-Mateo D. Lavín-Pérez A. M. Peñacoba C. Del Coso J. Leyton-Román M. Luque-Casado A.. (2021). Key factors associated with adherence to physical exercise in patients with chronic diseases and older adults: an umbrella review. Int. J. Environ. Res. Public Health 18:2023. doi: 10.3390/ijerph18042023, PMID: 33669679 PMC7922504

[ref8] Conversano C. Orrù G. Pozza A. Miccoli M. Ciacchini R. Marchi L.. (2021). Is mindfulness-based stress reduction effective for people with hypertension? A systematic review and Meta-analysis of 30 years of evidence. Int. J. Environ. Res. Public Health 18:2882. doi: 10.3390/ijerph18062882, PMID: 33799828 PMC8000213

[ref9] Cucher D. J. Kovacs M. S. Clark C. E. Hu C. K. (2023). Virtual reality consumer product injuries: an analysis of national emergency department data. Injury 54, 1396–1399. doi: 10.1016/j.injury.2023.01.030, PMID: 36803922

[ref10] Dzardanova E. Kasapakis V. (2022). Virtual reality: a journey from vision to commodity. IEEE Ann. Hist. Comput. 45, 18–30. doi: 10.1109/MAHC.2022.3208774

[ref11] Enkema M. C. McClain L. Bird E. R. Halvorson M. A. Larimer M. E. (2020). Associations between mindfulness and mental health outcomes: a systematic review of ecological momentary assessment research. Mindfulness 11, 2455–2469. doi: 10.1007/s12671-020-01442-2, PMID: 35694042 PMC9187214

[ref12] Fendel J. C. Bürkle J. J. Göritz A. S. (2021). Mindfulness-based interventions to reduce burnout and stress in physicians: a systematic review and meta-analysis. Acad. Med. 96, 751–764. doi: 10.1097/ACM.0000000000003936, PMID: 33496433

[ref13] Giulietti M. Spatuzzi R. Fabbietti P. Vespa A. (2023). Effects of mindfulness-based interventions (MBIs) in patients with early-stage Alzheimer’s disease: a pilot study. Brain Sci. 13:484. doi: 10.3390/brainsci13030484, PMID: 36979294 PMC10046197

[ref14] Gupta K. (2021). Mindfulness and meditation as pedagogical methods for adult and higher education. J. Contin. High. Educ. 69, 121–134. doi: 10.1080/07377363.2020.1825315

[ref15] Hendrixson H.A. (2020). Seniors and Mindfulness-Based Virtual Reality Experiences: Impacts on Quality of Life. Ball State University.

[ref16] Hsieh C.-C. Lin P.-S. Hsu W.-C. Wang J.-S. Huang Y.-C. Lim A.-Y.. (2019). The effectiveness of a virtual reality-based tai chi exercise on cognitive and physical function in older adults with cognitive impairment. Dement. Geriatr. Cogn. Disord. 46, 358–370. doi: 10.1159/000494659 30537752

[ref17] Huang S.-Y. Wang C.-Y. (2021). Product design investigation of Mobile phone VR headset. J Qual 28, 33–53. doi: 10.6220/joq.202102_28(1).0003

[ref18] Jamieson S. D. Tuckey M. R. (2017). Mindfulness interventions in the workplace: a critique of the current state of the literature. J. Occup. Health Psychol. 22, 180–193. doi: 10.1037/ocp0000048, PMID: 27643606

[ref19] Kim J. Kim Y. Chang P.-S. Min Oh S. Han S. (2022). A pilot study of virtual reality (VR) tai chi program on mental health among older adults during the COVID-19 pandemic. Am. J. Health Behav. 46, 576–585. doi: 10.5993/AJHB.46.5.8, PMID: 36333829

[ref20] Kim J. Son J. Ko N. Yoon B. (2013). Unsupervised virtual reality-based exercise program improves hip muscle strength and balance control in older adults: a pilot study. Arch. Phys. Med. Rehabil. 94, 937–943. doi: 10.1016/j.apmr.2012.12.010, PMID: 23262158

[ref21] Kriakous S. A. Elliott K. A. Lamers C. Owen R. (2021). The effectiveness of mindfulness-based stress reduction on the psychological functioning of healthcare professionals: a systematic review. Mindfulness 12, 1–28. doi: 10.1007/s12671-020-01500-9, PMID: 32989406 PMC7511255

[ref23] Lee B. M. Kim S.-W. Lee B. J. Won S.-H. Park Y. H. Kang C. Y.. (2023). Effects and safety of virtual reality-based mindfulness in patients with psychosis: a randomized controlled pilot study. Schizophrenia 9:57. doi: 10.1038/s41537-023-00391-8, PMID: 37704650 PMC10499950

[ref24] Li K. (2022). “Review of mindfulness uses, influencing factors and application” in 2022 8th International Conference on Humanities and Social Science Research (ICHSSR 2022) (Dordrecht: Atlantis Press), 540–546. doi: 10.2991/assehr.k.220504.098

[ref25] Li H. Zhang X. Wang H. Yang Z. Liu H. Cao Y.. (2021). Access to nature via virtual reality: a mini-review. Front. Psychol. 12:725288. doi: 10.3389/fpsyg.2021.725288, PMID: 34675840 PMC8523668

[ref26] Ma J. Zhao D. Xu N. Yang J. (2023). The effectiveness of immersive virtual reality (VR) based mindfulness training on improvement mental-health in adults: a narrative systematic review. Explore 19, 310–318. doi: 10.1016/j.explore.2022.08.001, PMID: 36002363

[ref27] May A. D. Maurin E. (2021). Calm: a review of the mindful meditation app for use in clinical practice. Fam. Syst. Health 39, 398–400. doi: 10.1037/fsh0000621 34410785

[ref28] Naude C. Skvarc D. Knowles S. Russell L. Evans S. Mikocka-Walus A. (2023). The effectiveness of mindfulness-based interventions in inflammatory bowel disease: a systematic review and meta-analysis. J. Psychosom. Res. 169:111232. doi: 10.1016/j.jpsychores.2023.111232 36990003

[ref29] Ngamkham S. Holden J. E. Smith E. L. (2019). A systematic review: mindfulness intervention for cancer-related pain. Asia Pac. J. Oncol. Nurs. 6, 161–169. doi: 10.4103/apjon.apjon_67_18, PMID: 30931361 PMC6371675

[ref30] Norman K. Haß U. Pirlich M. (2021). Malnutrition in older adults—recent advances and remaining challenges. Nutrients 13:2764. doi: 10.3390/nu13082764, PMID: 34444924 PMC8399049

[ref31] Odgers K. Dargue N. Creswell C. Jones M. P. Hudson J. L. (2020). The limited effect of mindfulness-based interventions on anxiety in children and adolescents: a meta-analysis. Clin. Child. Fam. Psychol. Rev. 23, 407–426. doi: 10.1007/s10567-020-00319-z, PMID: 32583200

[ref32] Padala K. P. Padala P. R. Lensing S. Y. Dennis R. A. Bopp M. M. Parkes C. M.. (2017). Efficacy of Wii-fit on static and dynamic balance in community dwelling older veterans: a randomized controlled pilot trial. J. Aging Res. 2017, 1–9. doi: 10.1155/2017/4653635 PMC5316445 28261500

[ref33] Rahman F. (2023). The Feasibility, Acceptability, and Usability of Seated Tai Chi Exergame among Frail Older Adult with Mild Dementia or Parkinson’s Disease: A Pilot Study. Ontario Tech University.

[ref34] Reangsing C. Rittiwong T. Schneider J. K. (2021). Effects of mindfulness meditation interventions on depression in older adults: a meta-analysis. Aging Ment. Health 25, 1181–1190. doi: 10.1080/13607863.2020.1793901, PMID: 32666805

[ref35] Sarkar T. D. Edwards R. R. Baker N. (2022). The feasibility and effectiveness of virtual reality meditation on reducing chronic pain for older adults with knee osteoarthritis. Pain Pract. 22, 631–641. doi: 10.1111/papr.13144, PMID: 35750655

[ref36] Scheepers R. A. Emke H. Epstein R. M. Lombarts K. M. (2020). The impact of mindfulness-based interventions on doctors’ well-being and performance: a systematic review. Med. Educ. 54, 138–149. doi: 10.1111/medu.14020, PMID: 31868262 PMC7003865

[ref37] Seabrook E. Kelly R. Foley F. Theiler S. Thomas N. Wadley G.. (2020). Understanding how virtual reality can support mindfulness practice: mixed methods study. J. Med. Internet Res. 22:e16106. doi: 10.2196/16106, PMID: 32186519 PMC7113800

[ref38] Sevinc V. Berkman M. I. (2020). Psychometric evaluation of simulator sickness questionnaire and its variants as a measure of cybersickness in consumer virtual environments. Appl. Ergon. 82:102958. doi: 10.1016/j.apergo.2019.102958, PMID: 31563798

[ref39] Trueba A. Crespo-Andrade C. Merino C. A. Frank N. A. A. Garces M. S. Cray H. V.. (2023a). Implementing affordable virtual reality interventions for older adults in Latin America: a feasibility study. Am. J. Geriatr. Psychiatry 31, S134–S135. doi: 10.1016/j.jagp.2022.12.185

[ref40] Trueba A. Park S. Dickinson R. Cray H. V. Kimball J. Vahia I. V. (2023b). Developing low-cost and scalable virtual reality mindfulness interventions for older psychiatry inpatients. Am. J. Geriatr. Psychiatry 31:S132. doi: 10.1016/j.jagp.2022.12.182

[ref41] Valikhani A. Kashani V. O. Rahmanian M. Sattarian R. Rahmati Kankat L. Mills P. J. (2020). Examining the mediating role of perceived stress in the relationship between mindfulness and quality of life and mental health: testing the mindfulness stress buffering model. Anxiety Stress Coping 33, 311–325. doi: 10.1080/10615806.2020.1723006, PMID: 32026721

[ref42] Wang X. Liang X. Yao J. Wang T. Feng J. (2023). A study of the use of virtual reality headsets in Chinese adolescents with intellectual disability. Int. J. Dev. Disabil. 69, 524–532. doi: 10.1080/20473869.2021.1970938, PMID: 37346261 PMC10281426

[ref43] Weber M. Schnorr T. Morat M. Morat T. Donath L. (2020). Effects of mind–body interventions involving meditative movements on quality of life, depressive symptoms, fear of falling and sleep quality in older adults: a systematic review with meta-analysis. Int. J. Environ. Res. Public Health 17:6556. doi: 10.3390/ijerph17186556, PMID: 32916879 PMC7559727

[ref44] Wu J. Ma Y. Zuo Y. Zheng K. Zhou Z. Qin Y.. (2022). Effects of mindfulness exercise guided by a smartphone app on negative emotions and stress in non-clinical populations: a systematic review and meta-analysis. Front. Public Health 9:773296. doi: 10.3389/fpubh.2021.773296, PMID: 35155341 PMC8825782

[ref45] Wu C. Yi Q. Zheng X. Cui S. Chen B. Lu L.. (2019). Effects of mind-body exercises on cognitive function in older adults: a meta-analysis. J. Am. Geriatr. Soc. 67, 749–758. doi: 10.1111/jgs.15714, PMID: 30565212

[ref46] Zenebe Y. Akele B. Necho M. (2021). Prevalence and determinants of depression among old age: a systematic review and meta-analysis. Ann. General Psychiatry 20, 1–19. doi: 10.1186/s12991-021-00375-x PMC8684627 34922595

[ref47] Zhang D. Lee E. K. Mak E. C. Ho C. Wong S. Y. (2021). Mindfulness-based interventions: an overall review. Br. Med. Bull. 138, 41–57. doi: 10.1093/bmb/ldab005, PMID: 33884400 PMC8083197

[ref48] Zhang X. Li H. Feng S. Su S. (2023a). The effect of velocity-based training variables on muscle strength: dose-response meta-analysis. Int. J. Sports Med. 44, 857–864. doi: 10.1055/a-2095-8254 37196672

[ref49] Zhang X. Zhang X. Feng S. Li H. (2023b). The causal effect of physical activity intensity on COVID-19 susceptibility, hospitalization, and severity: evidence from a mendelian randomization study. Front. Physiol. 14:1089637. doi: 10.3389/fphys.2023.1089637, PMID: 36969605 PMC10030504

[ref50] Zhao Y. C. Zhao M. Song S. (2022). Online health information seeking behaviors among older adults: systematic scoping review. J. Med. Internet Res. 24:e34790. doi: 10.2196/34790, PMID: 35171099 PMC8892316

[ref51] Zou L. Sasaki J. E. Wei G.-X. Huang T. Yeung A. S. Neto O. B.. (2018). Effects of mind–body exercises (tai chi/yoga) on heart rate variability parameters and perceived stress: a systematic review with meta-analysis of randomized controlled trials. J. Clin. Med. 7:404. doi: 10.3390/jcm7110404, PMID: 30384420 PMC6262541

